# A Mobile Robot Localization via Indoor Fixed Remote Surveillance Cameras †

**DOI:** 10.3390/s16020195

**Published:** 2016-02-04

**Authors:** Jae Hong Shim, Young Im Cho

**Affiliations:** 1 Department of Mechatronics Engineering, Korea Polytechnic University, Si-Heung, Gyunggi-do 429-793, Korea; jhshim@kpu.ac.kr; 2 Department of Computer Engineering, Gachon University, Sung-Nam, Gyunggi-do 461-701, Korea

**Keywords:** localization, mobile robot, surveillance camera, indoor, homography

## Abstract

Localization, which is a technique required by service robots to operate indoors, has been studied in various ways. Most localization techniques have the robot measure environmental information to obtain location information; however, this is a high-cost option because it uses extensive equipment and complicates robot development. If an external device is used to determine a robot’s location and transmit this information to the robot, the cost of internal equipment required for location recognition can be reduced. This will simplify robot development. Thus, this study presents an effective method to control robots by obtaining their location information using a map constructed by visual information from surveillance cameras installed indoors. With only a single image of an object, it is difficult to gauge its size due to occlusion. Therefore, we propose a localization method using several neighboring surveillance cameras. A two-dimensional map containing robot and object position information is constructed using images of the cameras. The concept of this technique is based on modeling the four edges of the projected image of the field of coverage of the camera and an image processing algorithm of the finding object’s center for enhancing the location estimation of objects of interest.  We experimentally demonstrate the effectiveness of the proposed method by analyzing the resulting movement of a robot in response to the location information obtained from the two-dimensional map. The accuracy of the multi-camera setup was measured in advance.

## 1. Introduction

Recently, various localization technologies for mobile robots have been studied with respect to acquiring accurate environmental information. Typically, mobile robots have self-organized sensors to obtain environmental information. However, such robots are very expensive to manufacture and have complicated body structures. The structural layout of indoor environments is typically a known state; thus, many localization studies have obtained the required information from external sensors installed on a robot. The infrared light, ultrasonic, laser range finder, RFID (Radio Frequency Identification), and RADAR (Radio Detecting and Ranging) are the popularly used sensors for localization. Hopper *et al.* [1] presented an active sensing system, which uses infrared emitters and detectors to achieve 5–10 m accuracy. However, this sensor system is not suitable for high-speed application as the localization cycle requires about 15 s and always requires line of sight. Ultrasonic sensor uses the time of flight measurement technique to provide location information [2]. However, the ultrasonic sensor requires a great deal of infrastructure for its high effectiveness and accuracy. Laser distance measurement is executed by measuring the time that it takes for a laser light to be reflected off a target and returned back to the sensor. Since the laser finder is a very accurate and quick measurement device, this device is widely used in many applications. Subramanian *et al.* [3] and Barawid *et al.* [4] proposed a localization method based on a laser finder. The laser finder was used to acquire environment distance information that can be used to identify and avoid obstacles during navigation. However, their high performance relies on high hardware costs. Miller *et al.* [5] presented an indoor localization system based on RFID. RFID based localization used RF tags and a reader with an antenna to locate objects, but detection of each tag can only work over about 4–6 m. Bahl *et al.* [6] and Lin *et al.* [7] introduced the RADAR system, which is a radio-frequency (RF) based system for locating and tracking users inside buildings. The concept of RADAR is to measure signal strength information at multiple stations positioned to provide overlapping field of coverage. It aggregates real measurements with signal propagation modeling to determine object location, thereby enabling location-aware applications. The accuracy of the RADAR system was reported by 2–3 m. 

Recently, visual image location systems have been preferred because they are not easily disturbed by other sensors [2,8,9]. Sungho [10] used workspace landmark features as external reference sources. However, this method has insufficient accuracy and is difficult to install and maintain due to the required additional equipment. Kim *et al.* [11] proposed the augmented reality techniques to achieve an average location recognition success rate of 89%, though the extra cost must be considered. Cheoket *et al.* [12] developed a method of localization and navigation in wide indoor areas by using a vision sensor. Though the set-up cost is lower, this method is not easy to implement if users do not have knowledge about the basic concept of electronic circuit analysis Recently, two camera localization systems have been proposed [13,14]. The concept of this system is that the object distances can be calculated by a triangular relationship from two different images of the cameras. However, to ensure the measuring reliability, the relative coordinates between two cameras must be maintained at the same position. In addition, the set-up cost of the experimental environment is quite expensive due to the use of two cameras. 

Nowadays, surveillance systems exist in most modern buildings, and cheap cameras are usually installed around these buildings. Indoor surveillance cameras are typically installed without blind areas, and visual data are transferred to a central data server for processing and analysis. If a mobile robot can determine its position using indoor cameras, it would not require an additional sensor for localization and could be applied to multi-agent mobile robot systems [15,16]. Kuscue *et al.* [17] and Li *et al.* [18] proposed a vision-based localization method using a single ceiling mounted surveillance camera. However, there are several problems that must be addressed prior to the realization of this concept. First, lens distortion arises from the poor-quality lenses in surveillance cameras, and shadow effects are produced by indoor light sources [19,20]. Second, information about occluding objects cannot be obtained using a single camera. Third, calibrations of camera and a map for localization are carried out independently, and it is very time-consuming work. 

Herein, we propose a localization method for a mobile robot to overcome the abovementioned problems associated with indoor environments. A two-dimensional map containing robot and object position information is constructed using several neighboring surveillance cameras [21]. The concept of this technique is based on modeling the four edges of the projected image of the field of coverage of the camera and an image processing algorithm of the finding object’s center for enhancing the location estimation of objects of interest. This approach relies on coordinate mapping techniques to identify the robot in the environment using multiple ceiling-mounted cameras. It can be applied for localization in complex indoor environments like T- and L-shaped environments. In addition, simultaneous calibration of cameras and a two-dimensional map can be carried out. Via the above modeling process, a 2D map is built in the form of air-view and quite accurate location can be dynamically acquired from a scaled grid of the map. Significant advantages of the proposed localization are its minimal cost, simple calibration and little occlusion, where it needs multiple ceiling-mounted inexpensive cameras that are installed in opposition to each other and wirelessly communicate with the mobile robot and update its current estimated position. Moreover, we experimentally demonstrate the effectiveness of the proposed method by analyzing the resulting robot movements in response to the location information acquired from the generated map.

## 2. Two-Dimensional Visual Map by Using the Homograph

### 2.1. Projected Image Plane for Two Dimensional Map

**Figure 1:**
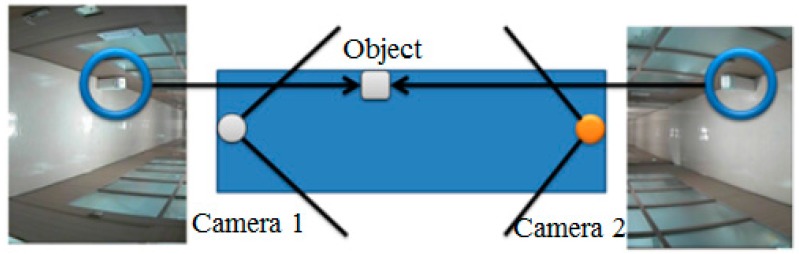
Images viewed from two neighboring indoor surveillance cameras.

Indoor surveillance cameras are typically installed to view the same object from opposite directions. Such images contain ground-area information that may be occluded by objects, as shown in Figure 1. Therefore, two object images viewed from opposite directions must be combined into a single image. We have attempted to accomplish this using homography. 

**Figure 2:**
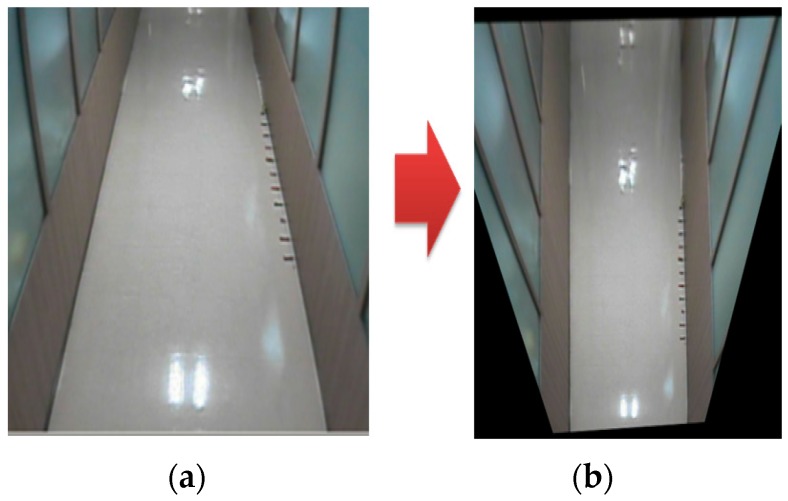
(**a**) Original image; (**b**) Air view image of (**a**).

Homography is a projection wherein a plane is transformed into another plane in space. A surveillance camera is mounted on a slant to obtain an image, as shown in Figure 2a. To observe the position and size of an object viewed from the camera, the image in Figure 2a is transformed into the air-view image of Figure 2b using homography.

**Figure 3:**
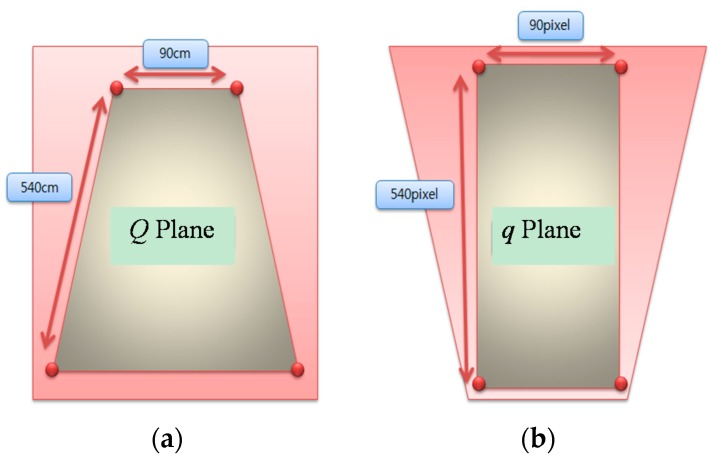
(**a**) Q plane of the original image; (**b**) q plane of the air view image of (**a**).

To transform an original image from a surveillance camera into an air-view image, a feature point *Q* of the original image is matched with the corresponding point *q* of the air-view image, as shown in Figure 3. We used a large placard of a chess board to match feature points between the original and air-view images.

Using Equation (1), a homography matrix *H* is obtained using four points from both plane *Q* and plane *q*

(1) q=HQ

The resulting homographically transformed positions of the same feature points of these two images from two surveillance cameras are combined in a new projected plane, as shown in Figure 4①. This results in a single united plane, as shown in Figure 4②. We can then construct a two-dimensional map, as shown in Figure 4③, by extracting the region of interest (ROI) of the actual floor area from the single plane. The homography transformation process makes the floor width of the projected image to be spread evenly like the air-view image. At that time, the distortion of the camera could be compensated together. 

**Figure 4:**
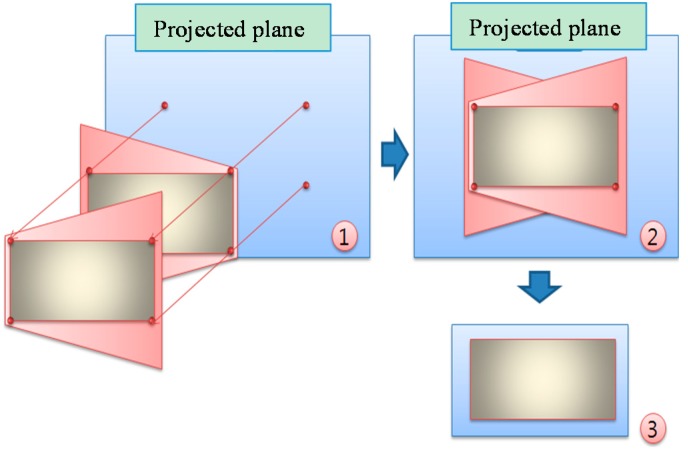
Extracting the two-dimensional map from two projected images by homography. ① Two images on the projected plane; ② A single united plane; ③ An extracted two dimensional map.

If multiple cameras are used for localization in more complex indoor environments, rotational relationships between image planes of the cameras are considered as shown in Equation (3).

(2) $$q=\;\left[\begin{array}{ccc} n_{x} & o_{x} & a_{x}\\ n_{y} & o_{y} & a_{y}\\ n_{z} & o_{z} & a_{z} \end{array}\right]HQ$$

where $\bar{n}=\;\lfloor \begin{array}{ccc} n_{x} & n_{y} & n_{z} \end{array}\rfloor$, $\bar{o}=\;\lfloor \begin{array}{ccc} o_{x} & o_{y} & o_{z} \end{array}\rfloor$, $\bar{a}=\;\lfloor \begin{array}{ccc} a_{x} & a_{y} & a_{z} \end{array}\rfloor$ are normal, orientation and approach unit vectors, respectively. 

### 2.2. Object Modeling on the Two-Dimensional Map

Here, we present a method to acquire the position and size of an object image on the two-dimensional map. Since two neighboring cameras view an object from opposite directions, their images of the same object differ. However, the floor area occupied by the object is the same in the two images. Therefore, if the rest of the image (except for the floor area) is deleted, we can obtain the actual floor area of the object on the two-dimensional map. Even if an object is looked at from opposite directions by two cameras, image correspondence on the two-dimensional map can be obtained by adopting area features of the object, *i.e.*, center position and size of the area. 

**Figure 5:**
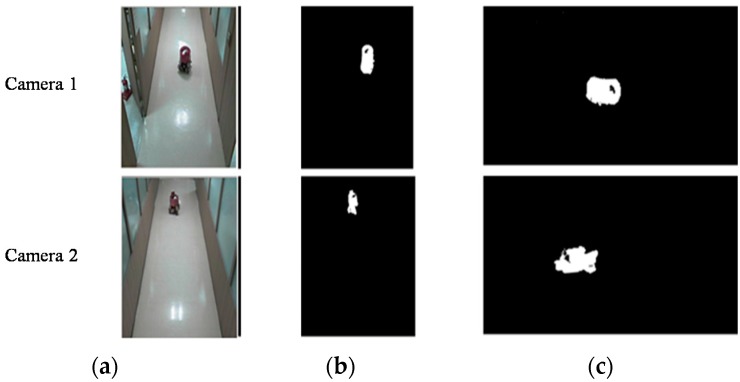
Projected images of two surveillance cameras by homography. (**a**) Original images; (**b**) Binary images; (**c**) Projected images.

To obtain the object floor area on the two-dimensional map, two projected images are transformed from their original images, as shown in Figure 5. Figure 5a shows the original images from the two cameras, Figure 5b shows binary images of Figure 5a with shadow effects removed, and Figure 5c shows the projected images of Figure 5b obtained by homography. If the coordinates of the two projected images are similar, the actual size and position of an object in contact with the floor, as represented by the image, are nearly the same. If the rest of the images not including the floor area are eliminated, the object image can be expressed with an air view. Equation (3) represents the size and position of an object on the projected plane *H*(*x*, *y*).

(3) $$H(x,y)\;=\;\{\;\begin{array}{cc} 1, & \;if\left\lfloor I_{1}^{H}(x,y)\text{​ }\&\;I_{2}^{H}(x,\text{​ }y)\right\rfloor \;=\;1\\ 0 & otherwise \end{array}$$

where $I_{1}^{H}(x,y)$ and $I_{2}^{H}(x,y)$ are the projected images of cameras 1 and 2, respectively. 

**Figure 6:**
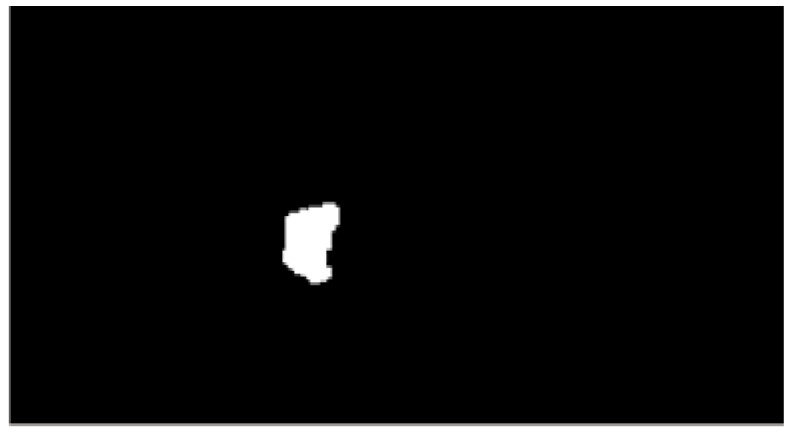
Common area of the projected images of cameras 1 and 2 in Figure 4③.

The common area between cameras 1 and 2, as shown in Figure 5c, can be presented as shown in Figure 6. The area is the object image on the two-dimensional map based on homography. 

Now, the size and center position of the object image on the two-dimensional map can be calculated. Generally, labeling or a contour technique is used to detect the area shape of an object in the image. These techniques are suitable for detecting the area shape from an image, such as Figure 6, which is a binary image. We apply the contour technique to rapidly determine its size and center position. Then, the moment of the area shape is calculated to obtain its center point and area. The moment is used to measure the size of the area shape. We obtain the size and center position of the object area from the edge information via the abovementioned process. 

**Figure 7:**
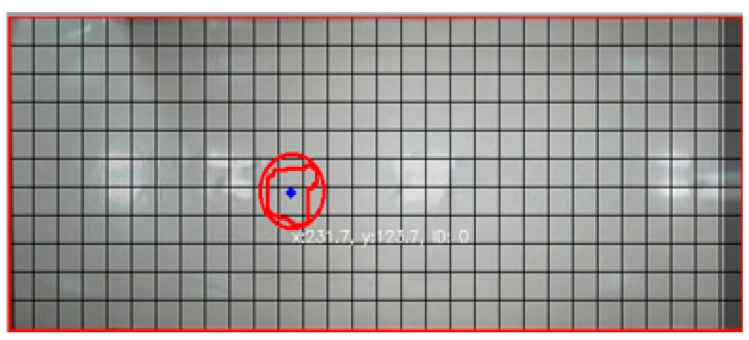
Two-dimensional map combined with the object area and the projected floor image.

To compare the object area with its real position, the actual floor image is transformed onto a projected plane and the object area is detected by contour processing, as shown in Figure 7.

### 2.3. Calibration of the Detected Object Area

**Figure 8:**
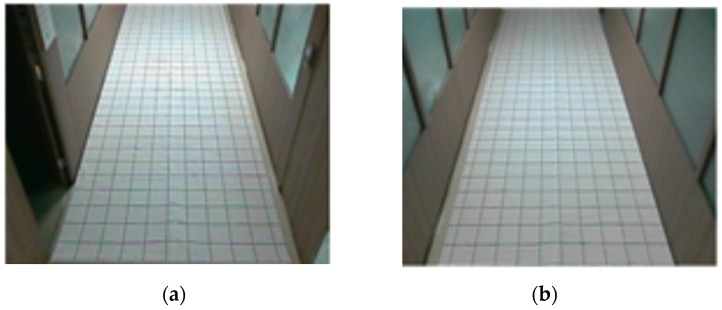
Grid placard for measuring the position error of the visually detected object area (**a**) camera 1; (**b**) camera 2.

When the size and center position of the object area obtained from contour processing are compared to the actual values of the object, considerable errors are revealed. We then measure the difference between the position of the real and visually detected object using a measurement grid, as shown in Figure 8. 

**Figure 9:**
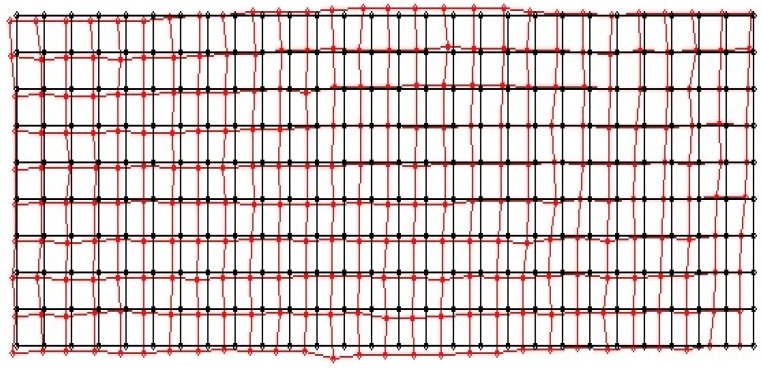
First experimental results of position errors between the real and the visually detected center position of the object.

In a calibration experiment, we used a cylindrical object (diameter, 20 cm). Figure 9 shows position errors between the real (black line) and visually detected center position (red line) of the object. 

**Figure 10:**
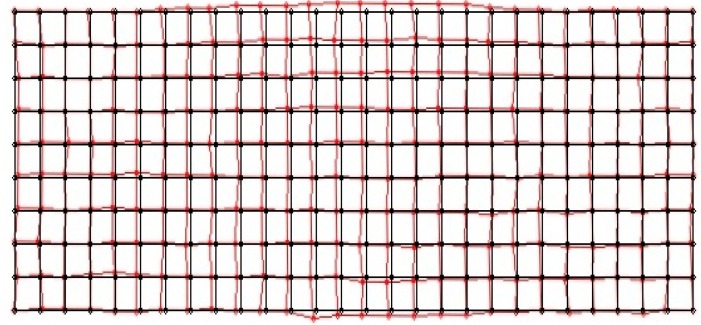
Error compensation results for the object area detected by homography.

There is considerable difference between the real and visually detected center position of the object, as shown in Figure 9. We consider that the error is caused by scale changes in the image projection and the installation error of the measurement grid. After the initial measurement to detect the object area, error compensation was performed using homography. When the visually detected grids were mapped to the floor image in an air view, we obtained the position with an error bound of 7.1 cm on the two-dimensional map. Figure 10 shows a representation of the error-compensated results of the object area, as detected by homography.

## 3. Topology Map Building and Optimal Path

Path planning is required for a robot to safely move without colliding into any object placed on the two dimensional map (Section 2). Herein, we employ the thinning algorithm [22]. The thinning algorithm leaves a single pixel in the center after continuous elimination of contour in random areas. Thus, a path by which a robot can avoid obstacles and move safely is generated by the thinning algorithm. 

**Figure 11:**
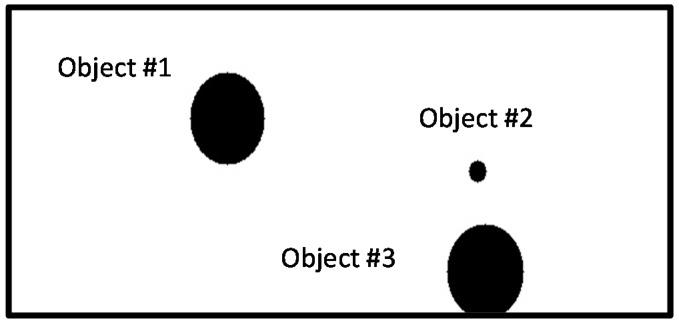
Example image of objects on a two-dimensional map before applying the thinning algorithm.

**Figure 12:**
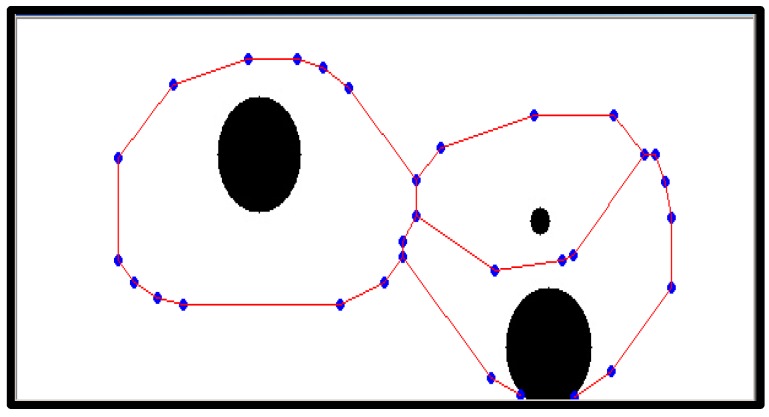
Topology map after applying the thinning algorithm.

After generating a moving path with the thinning algorithm, as shown in Figure 11 and Figure 12, a movement indicator is required for a robot. Thus, a topological map is generated to create the path indicator. However, the algorithm can generate a path that is difficult for a robot to move along; thus, eliminating information about such paths by checking the area around nodes is required. To make robots move through nodes, an area around each node that is larger than that of the robot should be examined to eliminate nodes and edges that cannot be traversed by the robot. Note that a robot cannot pass if there is an object in the search field around a node and if the search field exists beyond the boundary of an image around a node.

**Figure 13:**
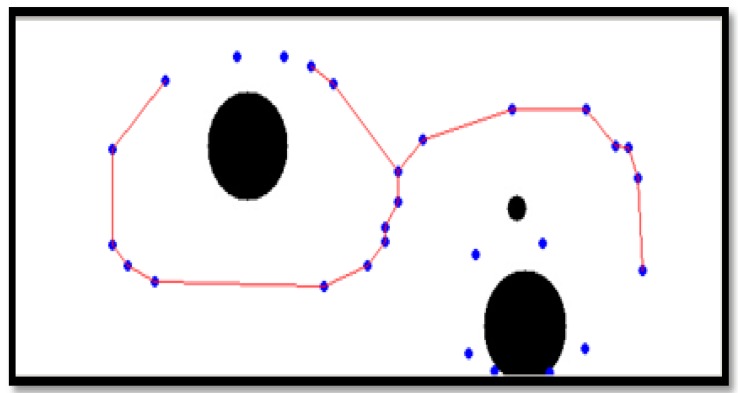
Modified driving path via searching path.

Figure 13 shows an image formed after removing a searched path by which a robot cannot pass. A path by which a robot could pass was generated with a topology map by applying the thinning algorithm. 

The A* algorithm is a graph exploring algorithm that calculates an optimal driving path with a given starting point and goal [23]. It uses a heuristic estimate on each node to estimate the shortest route to the target node with minimal calculation. 

**Figure 14:**
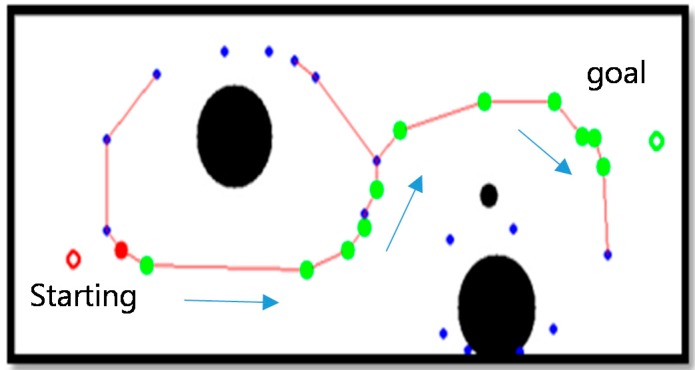
Shortest robot path by A* algorithm.

Figure 14 shows the shortest robot path estimated by the A* algorithm. A robot moves along nodes on the estimated path.

## 4. Experimental Results

We performed a series of experiments to demonstrate the effectiveness of the proposed two-dimensional-map-based localization method using indoor surveillance cameras. The width and length of the floor viewed by the two neighboring cameras were 2.2 and 6 m, respectively. The detected two-dimensional map by homography represents the area of the floor viewed by the two cameras in an air view. We used a self-developed mobile robot with an omnidirectional wheel in the experiment. The surveillance camera had a resolution of 320 × 240 pixels and three RGB (Red Green Blue) channels. 

**Figure 15:**
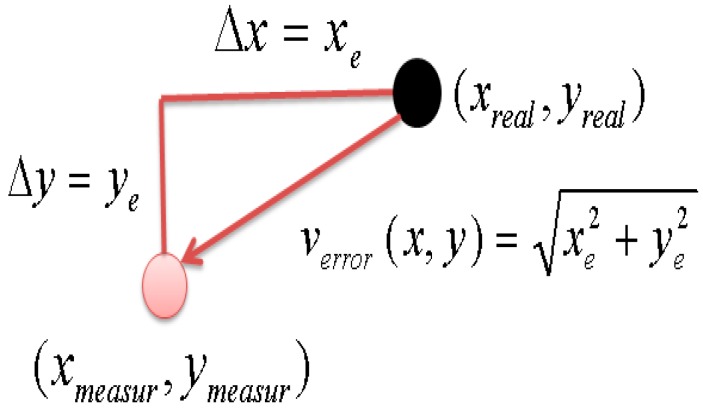
Position error estimate.

**Figure 16:**
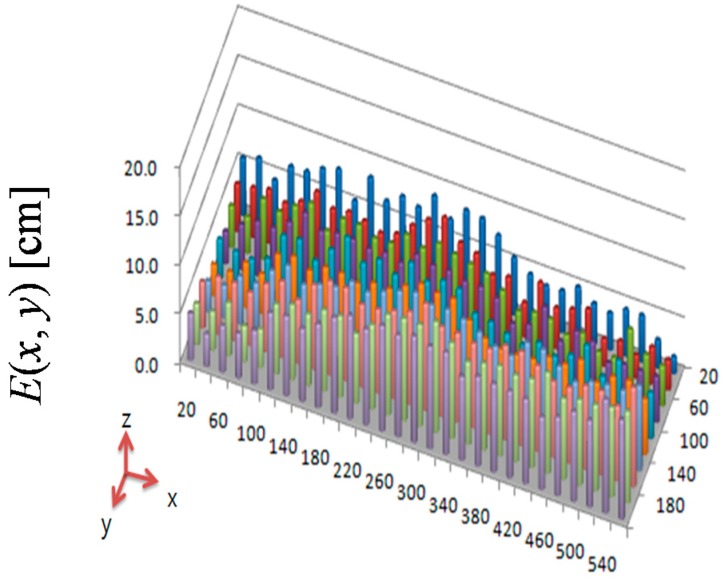
Position error estimate before error compensation.

The accuracy of the two dimensional map with the proposed method was experimentally obtained. Each position error in Figure 15 has two *x*- and *y*-axis components in a plane. To represent two error components as a single parameter at each position, we suggest the position error estimation shown in Figure 16. Here, *x*_real_ and *y*_real_ mean the real position, and *x*_measur_ and *y*_measur_ represent the visually detected object position on the two dimensional map. *x*_e_ and *y*_e_ are obtained using Equation (4).

(4) $$x_{e}=x_{real}-x_{measur}\;,\;\;\;\;\;y_{e}=\;y_{real}-\;y_{measur}$$

Position error *v*_error_ is composed of *x*_e_ and *y*_e_ in Equation (4) and is expressed by Equation (5). The magnitude of position error estimate *v*_error_ is obtained using Equation (6).

(5) $$v_{error}\left(x,\;y\right)=\;\left[x_{e},\;y_{e}\right]$$

(6) $$\left|v_{error}\left(x,\;y\right)\right|=\;\sqrt{x_{e}^{2}+\;y_{e}^{2}}$$

We define the position error estimate *E*(*x*, *y*) as the absolute value of *v*_error_ using Equation (7).

(7) $$E\left(x,\;y\right)=\;\left|v_{error}\left(x,\;y\right)\right|$$

**Figure 17:**
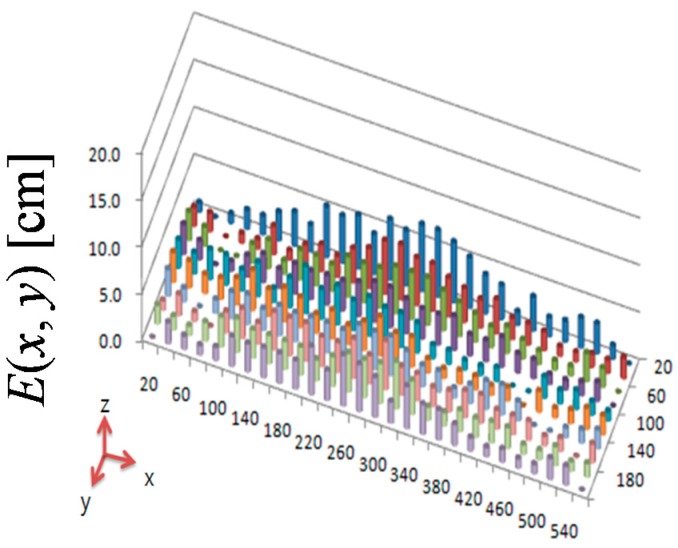
Position error estimate after error compensation.

Here, *E*(*x*,*y*) is the error plane of the difference between the real and visually detected object position. To examine the effectiveness of the proposed error compensation by homography (Section 2), we compared *E*(*x*,*y*) before and after error compensation. The position error estimate before error compensation is shown in Figure 16. The *x*–*y* plane of Figure 16 is the *x*–*y* plane of the two-dimensional map, and the *z* plane is the value of *E*(*x*, *y*). The maximum and average values of *E*(*x*, *y*) are 11.5 and 6.7 cm, respectively. After error compensation by homography, the maximum and average values of *E*(*x*, *y*) are 7.1 and 2.6 cm, respectively, as shown in Figure 17. The maximum position error was decreased by 38% through the proposed error compensation method. The accuracy of the two-dimensional map obtained by two ceiling surveillance cameras is 7.1 cm, which is sufficient for mobile robot localization.

**Figure 18:**
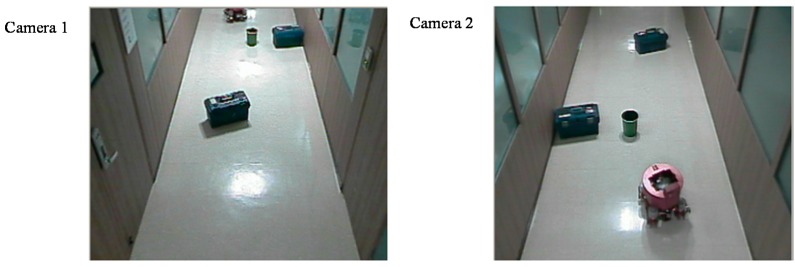
Objects in the experimental environment.

**Figure 19:**
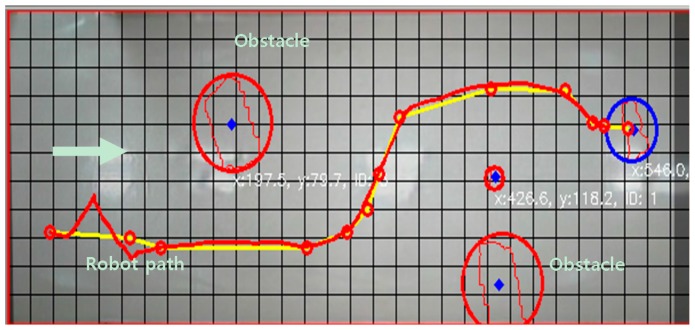
Experimental results of the robot’s path control using the proposed localization method.

Figure 18 shows two images from the two neighboring surveillance cameras. There are several objects on the floor. A mobile robot was controlled to move from one position to the opposite position using the proposed localization based on the two-dimensional map described in Section 2.2. We used the A* algorithm as the path planning method for the mobile robot. The objects on the floor were detected by homography as the object area in the projected plane, and the robot’s moving path was planned considering the object area in the two-dimensional map. The experimental results of the robot’s path control are shown in Figure 19. The error bounds between the planned and actual movement path of the robot was ±5 cm. This means that the proposed localization method may be effective for indoor mobile robots.

**Figure 20:**
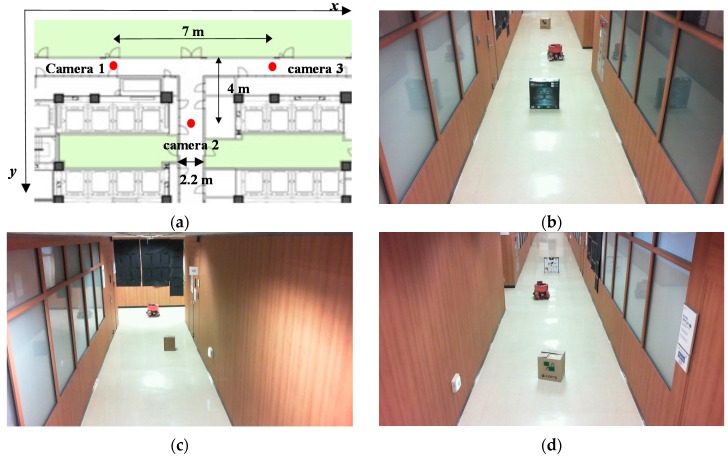
(**a**) Map of our experimental environment; (**b**) Image of camera 1; (**c**) Image of camera 2; (**d**) Image of camera 3.

To show that the proposed method can be applied for complex indoor environments, an experiment was carried out at a T-shaped indoor environment. As shown in Figure 20a, three surveillance cameras were used to build a two-dimensional map. Figure 20b–d show images from the three cameras. 

**Figure 21:**
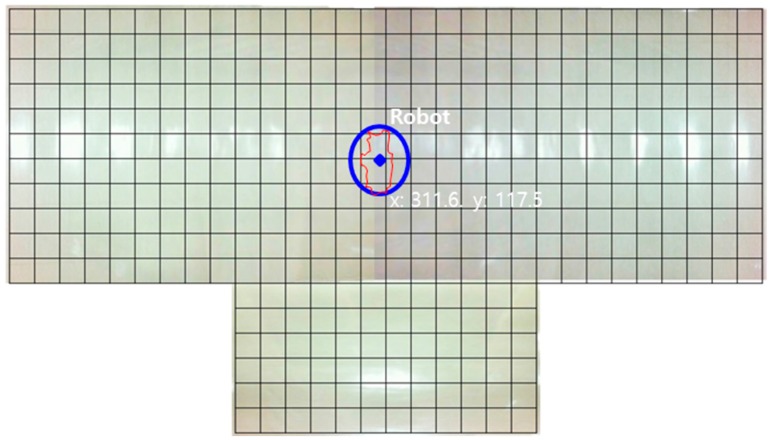
Experimental results of the two-dimensional map using the proposed localization method.

Figure 21 shows the two-dimensional map using the proposed method. The accuracy of the two-dimensional map by three ceiling surveillance cameras is 7 cm, which is satisfactory for mobile robot localization. 

## 5. Conclusions

We have proposed a new vision-based approach for mobile-robot localization in an indoor environment using multiple remote ceiling-mounted cameras. The proposed approach uses a two-dimensional mapping technique between camera and ground-image plane coordinate systems. We used homography to transform the image planes. Two camera-image planes were combined into a single ground-image plane with an air view, which resulted in a two-dimensional map. The position error bound of the developed two-dimensional map was within 7.1 cm. We performed a series of experiments to demonstrate the effectiveness of the proposed two-dimensional-map-based localization method. Among several obstacles fixed on the floor, the mobile robot successfully maneuvered to its destination position using only the two-dimensional map without the help of any other sensor. In future, we plan to extend the proposed method to the localization of multiple mobile robots in an indoor environment.
